# Defining characteristics and risk indicators for diagnosing nursing home-acquired pneumonia and aspiration pneumonia in nursing home residents, using the electronically-modified Delphi Method

**DOI:** 10.1186/s12877-016-0231-4

**Published:** 2016-03-07

**Authors:** Vanessa Hollaar, Claar van der Maarel-Wierink, Gert-Jan van der Putten, Wil van der Sanden, Bert de Swart, Cees de Baat

**Affiliations:** Department of Neurorehabilitation, HAN University of Applied Sciences, P.O. Box 6960, 6503 GL Nijmegen, The Netherlands; BENECOMO, Flemish-Netherlands Geriatric Oral Research Group, Ghent, Belgium; BENECOMO, Flemish-Netherlands Geriatric Oral Research Group, Nijmegen, The Netherlands; Center for Special Care in Dentistry, Gustav Mahlerlaan 3004, 1081 LA Amsterdam, The Netherlands; Department of Oral Function and Prosthetic Dentistry, Radboud university medical center, P.O. Box 9101, 6500 HB Nijmegen, The Netherlands; Amaris Gooizicht, Paulus van Loolaan 21, 1217 SH Hilversum, The Netherlands; Department of Rehabilitation, division Speech Therapy, Radboud university medical center, P.O. Box 9101, 6500 HB Nijmegen, The Netherlands

**Keywords:** Nursing home-acquired pneumonia, Aspiration pneumonia, Delphi method, Risk indicator, Characteristic, Nursing home

## Abstract

**Background:**

In nursing home residents, it is not possible to distinguish pneumonia and aspiration pneumonia clinically. International literature reveals no consensus on which and how many characteristics and risk indicators must be present to diagnose (nursing home-acquired) pneumonia and aspiration pneumonia. The aim of this survey was to reach consensus among a panel of clinical medical experts in geriatrics and pulmonology about the characteristics required for diagnosing pneumonia, and about the risk indicators needed to consider the diagnosis aspiration pneumonia in nursing home residents with pneumonia.

**Methods:**

Literature review and three expert-rating iterations using the electronically-modified Delphi Method were carried out. After each expert rating iteration, data analysis was performed. Qualitative responses and additional (nursing home-acquired) pneumonia characteristics which were mentioned in reply to structured open-ended questions were summarised, whilst similar responses were combined and these combinations were ordered by frequency in order to use them in the next iteration. Characteristics which failed to reach consensus were considered as inconclusive and eliminated. Consensus was reached when at least 70 % of the participants agreed.

**Results:**

Literature review revealed 16 currently used common characteristics for diagnosing (nursing home-acquired) pneumonia. No consensus was reached about characteristics and the number of characteristics required for diagnosing (nursing home-acquired) pneumonia. However, 57 % agreed that dyspnea, fever, deterioration of general functioning, tachypnea and crepitation with auscultation are the most important characteristics and the responses by the participants suggested that two or three characteristics should be present. Subsequently, 80 % of the participants agreed on the risk indicators dysphagia, choking incident, (history of) tube feeding, neurological disease and cognitive impairment for considering the diagnosis aspiration pneumonia in nursing home residents with pneumonia.

**Conclusions:**

No final consensus could be reached about which and how many characteristics are required for diagnosing pneumonia in nursing home residents. However, the results indicated that dyspnea, fever, deterioration of general functioning, tachypnea and crepitation with auscultation are characteristics of some importance and that at least two or three characteristics should be present. With regard to considering aspiration pneumonia in nursing home residents with pneumonia, final consensus was reached about the risk indicators dysphagia, choking incident, (history of) tube feeding, neurological disease and cognitive impairment.

## Background

Pneumonia is a serious lung infection and can be a life-threatening disease in nursing home residents [1]. The incidence of nursing home-acquired pneumonia (NHAP) is tenfold the incidence of community-acquired pneumonia (CAP) [2]. The mortality rate of NHAP is higher than the mortality rate of CAP, and NHAP is a leading cause of death in nursing home residents [3–5]. Less differences were found in the aetiology of NHAP and CAP [4, 6]. *Streptococcus pneumonia* is the most important pathogen in both pneumonias, whereas *Staphylococcus aureus* was frequently more present in NHAP than in CAP [4, 7]. Aspiration pneumonia is another specific type of pneumonia which frequently appears in nursing home residents due to the inhalation of colonised oropharyngeal material or gastric contents into the larynx and the lower respiratory tract [8].

In daily nursing home practice, it is difficult to diagnose (the three types of) pneumonia because it is not possible to distinguish the types clinically, and because specific markers are not available [9, 10]. Aspiration pneumonia in particular can be difficult to diagnose, because usually the moment of aspiration cannot be observed or the aspiration occurs “silently” [8]. Therefore, chest radiographic examination with fluoroscopy is needed to confirm the diagnosis of both pneumonia and aspiration pneumonia [8, 11]. However, chest radiographic examinations are seldom possible in nursing homes. Many nursing home residents suffer from terminal dementia. Consequently, they require appropriate care for their acute condition and specialized care for their chronic conditions. There is strong evidence that admission to a hospital results in a higher risk of iatrogenic complications [12, 13]. To prevent these complications, transferring a resident to a hospital to perform a chest radiographic is not recommended.

Therefore, it is of great concern to recognise characteristics of pneumonia early in order to prevent negative health outcomes. An early diagnosis of pneumonia will optimise the effects of treatment, reduce the complications and even reduce the risk of mortality [14, 15]. However, due to the atypical presentation of each type of pneumonia in nearly all nursing home residents, the (first) characteristics are not clearly recognised by physicians and nursing staff [1]. Furthermore, it is of great importance to recognise risk indicators which are involved in the development of the disease. An important risk factor of aspiration pneumonia is dysphagia [14, 17–20]. Other identified risk indicators are old age, male gender, lung diseases, diabetes mellitus, poor oral health, severe dementia, Parkinson’s disease, malnutrition, and the use of antipsychotic drugs, proton pump inhibitors and angiotensin-converting enzyme inhibitors [17, 21].

Up till now, the international literature has not revealed consensus about which and how many characteristics and risk indicators must be present in order to diagnose (nursing home-acquired) pneumonia and aspiration pneumonia. The aim of this survey was to reach consensus among a panel of clinical medical experts in geriatrics and pulmonology about characteristics required for diagnosing pneumonia and about risk indicators needed to consider the diagnosis aspiration pneumonia in nursing home residents with pneumonia.

## Methods

### Survey design: electronically-modified Delphi Method

The electronically-modified Delphi Method was carried out online among a panel of (highly) qualified clinical medical experts in geriatrics and pulmonology in The Netherlands. Originally, the Delphi Method was developed to predict the impact of technology on warfare [22]. Nowadays, the method has been modified to gather and structure information on a particular issue from experts in an electronical way, using an iterative process to reach consensus on that issue [23, 24]. A panel of experts responds to questions anonymously and receives feedback by a review of the responses by all panel members involved. This process is repeated until consensus is reached.

A heterogeneous panel of 15 to 30 participants and a homogeneous panel of 10 to 15 participants are deemed sufficient [25, 26]. No generally accepted criteria for selecting experts are available and neither are recommendations on the minimal or maximal number of participants [27]. The representativeness of the survey seems dependent on the qualities of the expert panel rather than on the number of participants [28]. Diversity of experts produces a greater variety of perspectives on a certain problem, more alternatives and more readily acceptable solutions than those of a homogeneous group [29, 30]. Advantages of the electronically-modified Delphi Method are the potential involvement of experts from diverse geographical regions and the mutual anonymity or faceless acting of the expert panel. Anonymity minimizes potential group pressure and dominance, as the latter could affect the validity of the outcome negatively [31].

Three iterations are usually sufficient to accomplish a high level of agreement about a certain issue and have proven to be sufficient for attaining stability in the responses [23, 31, 32].

The current survey was conducted between February 2012 and November 2013 in four Phases: a literature review of the currently used characteristics for diagnosing (nursing home-acquired) pneumonia (Phase 1), two general expert-rating iterations (Phase 2 and Phase 3) and a consensus expert-rating iteration (Phase 4).

### Expert panel

Given that no recommendations for the number of participants of an expert panel for the electronically-modified Delphi Method were available, initially as many as possible clinical medical experts in geriatrics or pulmonology were invited to participate. The medical experts in geriatrics who were selected, were members of a national scientific geriatric medicine association. Their email addresses were collected during member meetings. The other selected people were experts in geriatrics and pulmonology, according to a statement on the website of an (academic) medical centre in The Netherlands. Before the members of the expert panel completed the questionnaire, they were informed about this research project. This information was described in an email message, which contained a link to the questionnaire and by completing the questionnaire written informed consent was given.

The survey included an experimental research project among medical professionals and was not a medical research experiment among patients. This survey is not defined in the Netherlands as a medical research experiment, making approval from an ethics committee not required [33].

### Data collection

To perform this survey, the online programme ‘Survey Monkey’ was used to create and send the questionnaires online to the expert panel and to gather the questionnaire data. In a previous survey using the electronically-modified Delphi Method, this programme had served appropriately [34].

#### Phase 1

The literature was reviewed to search for currently used common characteristics of (nursing home-acquired) pneumonia with the objective to compose a list of characteristics which could be used in Phase 2.

#### Phase 2

In the first expert-rating iteration, the selected experts received an invitation to participate by email with a hyperlink to an online questionnaire. After 2 weeks, the experts received, by email, a reminder in case the questionnaire had not yet been completed.

The online questionnaire contained three multiple-choice questions and two structured open-ended questions. The multiple-choice questions were: ‘*What is your medical specialty?’, ’Which characteristics are the most important when diagnosing (nursing-home acquired) pneumonia?’,* and *‘How many characteristics must be present for diagnosing (nursing home-acquired) pneumonia?’.* With regard to the first question, the list of (nursing home-acquired) pneumonia characteristics derived from the literature review in Phase 1 was submitted, and the experts were requested to rate 10 characteristics in order of importance of presence by giving each of the 10 characteristics a numerical code from 1 (‘of no importance at all’) to 10 (‘of utmost importance’). The two structured open-ended questions were: *‘Which characteristics are lacking for diagnosing (nursing home-acquired) pneumonia?’ and ‘Which risk indicators must be present in nursing home residents with pneumonia to make you consider the diagnosis aspiration pneumonia?’.* The results were analysed and the list of (nursing home-acquired) pneumonia characteristics was adjusted accordingly.

#### Phases 3 and 4

In the second and third expert-rating iteration, the selected experts received an invitation to participate by email, with a hyperlink to the adjusted online questionnaire. After 2 weeks, they received, by email, a reminder in case the questionnaire had not yet been completed.

### Data analysis

The Statistical Package for the Social Sciences (SPSS) 20.0 (Chicago, IL, USA) was used for data analysis after each expert-rating iteration (Phases 2, 3 and 4). Qualitative responses to structured open-ended questions were summarized by the first author. Similar responses were combined and these combinations were ordered by frequency. In order to decide whether additional (nursing home-acquired) pneumonia characteristics mentioned in reply to the open-ended question were valuable for use in the next Phase, their frequency was registered. Characteristics which failed to reach consensus were considered as inconclusive and therefore eliminated. In Phases 3 and 4, the same procedure as the one that had been followed for the selection of characteristics for diagnosing (nursing home-acquired) pneumonia, was performed for the selection of risk indicators of aspiration pneumonia. Consensus was reached when a minimum of 70 % of the participants agreed.

## Results

### Phase 1: Literature review

The literature review was performed in December 2011 and revealed 16 currently used common characteristics for diagnosing (nursing home-acquired) pneumonia, which are listed in Table 1 [16, 35–37]. This list of characteristics was presented to the experts who were invited to participate in Phase 2.

**Table 1:** Common characteristics for diagnosing (nursing home-acquired) pneumonia, yielded by the literature review of Phase 1, and frequency of importance scores (1 = hardly important; 10 = most important), numbers of scores on the common characteristics, 50 % indications and frequency indications, all resulting from Phase 2

Characteristic	Frequency of importance score										Number of scores	50 % Indication as well as frequency indication
	1	2	3	4	5	6	7	8	9	10		
Tachypnea	1	4	2	5	7	10^b^	4	11^b^	6	15^b^	65^a^	^c^
Dyspnea	5	1	5	1	5	5	6^b^	9^b^	15^b^	13^b^	65^a^	^c^
Crepitation with auscultation	2	3	5	3	3	10^b^	8^b^	12^b^	2	7^b^	55^a^	^c^
Fever (temperature > 38 **°**C)	5	3	2	3	10	7	14^b^	9^b^	10^b^	6	69^a^	^c^
Coughing	2	6	2	6	7	7^b^	11^b^	8^b^	9^b^	6	64^a^	^c^
Deterioration of general functioning	7	2	6	7	9	3	5	9^b^	5	5	58^a^	^c^
Leucocystosis	3	3	7	8	5	4	6^b^	4	6^b^	0	46^a^	^c^
(Central) cyanosis	6	6	6	8	3	2	1	0	1	1	34	
Hypoxia	2	3	8	7	6	6^b^	5	6^b^	6^b^	4	53^a^	^c^
Pain of breathing and coughing	3	6	8	3	6	4^b^	2	1	1	2	36^a^	
Shivers	3	10	3	3	4	4	1	1	1	0	30	
Decreased consciousness	10	5	3	8	3	3	2	4	1	1	40^a^	
Purulent sputum	2	7	7	6	5	7^b^	6	5	3	3	51^a^	
Tachycardia	2	2	3	3	6	6	4	3	2	0	31	
Pleura rub	2	5	0	2	2	0	2	3	2	1	19	
Transpiration	3	4	5	3	3	0	0	0	0	0	18	

^a^ Selected by more than 50 % of the participants (50 % indication)

^b^ Included in the 4 highest frequency of importance scores, as far as present in the 5 highest importance scores (frequency indication)

^c^ Sorted out for Phase 3 because of the combination of 50 % indication and frequency indication

### Phase 2

Seventy of 214 initially invited experts agreed to participate in Phase 2 (33 %). Fifty-eight participants were elderly care physicians (83 %), eight geriatricians (10 %), two pulmonologists (3 %), one internist specialized in geriatrics (2 %) and one cardiologist specialised in geriatrics (2 %). In The Netherlands, elderly care physicians are medical specialists in health care for (frail and disabled) older people. They are not employed by hospitals, but often by nursing homes [38].

Table 1 shows, in the row “number of scores”, how frequently each characteristic was selected by the participants. The characteristics which were selected by more than 50 % of the participants, 36 or more, were indicated (50 % indication: #). Furthermore, the table shows the frequency of importance scores on the 16 characteristics presented to the participants. For each characteristic which had a 50 % indication (#), the four highest frequency of importance scores, as far as present in the five highest importance scores (6–10), was indicated (frequency indication: *). Characteristics were selected for Phase 3 when they had a 50 % indication (#) and at least one frequency indication (*) in the three highest importance scores or at least two frequency indications (*) in the five highest importance scores. This procedure implied that a characteristic was selected for Phase 3 when it was chosen by more than 50 % of the participants and when the participants had found its presence most important for diagnosing (nursing home-acquired) pneumonia. The eight characteristics selected in this way were tachypnea, dyspnea, crepitation with auscultation, fever, coughing, deterioration of general functioning, leucocytosis and hypoxia, as indicated in the Table 1.

According to 57 participants (81 %), some characteristics for diagnosing (nursing home-acquired) pneumonia were lacking in the list they were presented, but 13 participants (19 %) did not recommend any additional characteristic. Some additional characteristics were suggested, but these characteristics were not used for Phase 3 due to the restricted frequency of the suggestions.

Four participants (6 %) indicated that one characteristic must be present in order to diagnose (nursing home-acquired) pneumonia in nursing home residents, six (9 %) indicated two, 29 (41 %) indicated three, 25 (36 %) indicated four, five (7 %) indicated five and one (1 %) indicated that six characteristics must be present. The mean number and standard deviation of characteristics indicated to be present for diagnosing (nursing home-acquired) pneumonia was 3.34 ± 0.99. Therefore, it was decided to use the number of three characteristics.

The participants mentioned that in case of (nursing home-acquired) pneumonia, they considered the diagnosis aspiration pneumonia when the following risk indicators were present: dysphagia, choking incident, (history) of tube feeding, neurological disease, decreased consciousness, dementia, delirium and cognitive impairment. Decreased consciousness, dementia, delirium and cognitive impairment were bundled as one risk factor because only few participants mentioned those and because they are indicators for cognitive confusion or deterioration. The bundle of risk indicators was indicated as cognitive impairment. The frequency of the risk indicators mentioned by the participants was as follows: dysphagia (60); choking incident (31); (history of) tube feeding (17); neurological disease (10) and cognitive impairment (24).

The questionnaire as well as the list with (nursing home-acquired) pneumonia characteristics used in Phase 2, were adjusted for Phase 3, concurrently with the results of Phase 2.

### Phase 3

The adjusted questionnaire contained eight questions, five multiple-choice questions and three structured open-ended questions, including the according to the results of Phase 2 adjusted list with characteristics for diagnosing (nursing home-acquired) pneumonia and a list with risk indicators for considering the diagnosis aspiration pneumonia (Tables 2 and 3). The multiple-choice questions were: ‘*What is your medical specialty?’, ‘Please select from the list provided the four most important characteristics for diagnosing pneumonia in nursing home residents in order of importance (4 = most important)’ and ‘Please select in the list provided the risk indicators that make you consider the diagnosis aspiration pneumonia in nursing home residents with pneumonia in order of importance (5 = most important)’.* The structured open-ended questions were related to gender, age and working experience of the participants and to (nursing home-acquired) pneumonia characteristics and aspiration pneumonia risk indicators which were lacking*.*

**Table 2:** Characteristics for diagnosing (nursing home-acquired) pneumonia, derived from Phase 2, and frequency of importance scores (4 = most important), number of scores on the characteristics and 50 % indications (#), all resulting from Phase 3

Characteristics	Frequency of importance score				Number of scores
	1	2	3	4	
Dyspnea	9	5	11	12	37#
Fever (temp >38 °C)	5	8	13	5	31#
Deterioration of general functioning	5	10	6	8	29#
Tachypnea	1	5	8	11	25#
Crepitation with auscultation	3	8	4	8	23#
Coughing	7	4	7	3	21
Leucocystosis	0	7	4	2	13
Hypoxia	2	2	3	4	11

**Table 3:** Risk indicators of aspiration pneumonia in nursing home residents with pneumonia, derived from Phase 2, and frequency of importance scores (5 = most important) and numbers of scores of the risk indicators, resulting from Phase 3

Risk indicators	Frequency of importance score					Number of scores
	1	2	3	4	5	
Dysphagia	3	4	1	15	21	44
Choking incident	3	5	9	8	16	41
(History) of tube feeding	9	8	18	3	5	43
Neurological disease	7	13	10	11	3	44
Cognitive impairment	15	7	11	7	4	44

The 70 participants of phase 2 were invited to participate. Forty-four (63 %) of them responded: 23 men (52 %) with an average age of 52.7 ± 6.1 years and 21 women with an average age of 48.4 ± 7.4 years, 38 elderly care physicians (86 %), 4 geriatricians (9 %) and 2 pulmonologists (5 %). These participants had an average working experience of 20.7 ± 8.4 years.

Despite the instruction to select the four most important characteristics for diagnosing (nursing home-acquired) pneumonia, 14 participants selected five characteristics. Table 2 shows, in the row “number of scores”, how frequently each characteristic was selected by the participants. The characteristics which were selected by more than 50 % of the participants, 23 or more, were indicated (50 % indication: #) and chosen for Phase 4. These five characteristics were dyspnea, fever, deterioration of general functioning, tachypnea and crepitation with auscultation. Some additional characteristics were suggested, but these characteristics were not used for Phase 4 due to the restricted frequency of the suggestions.

Table 3 shows, in the row “number of scores”, how frequently each risk factor for considering the diagnosis aspiration pneumonia was selected by the participants. Unfortunately, three participants had not selected each risk factor in order of importance leading to two risk indicators with “number of scores” lower than 44. Furthermore, the table shows the frequency of importance scores of the five risk indicators presented to the participants. Each risk factor had, for the three highest importance scores (3–5), a total score of 50 % or higher. This means that half or more of the participants had rated all risk indicators which were important for considering the diagnosis aspiration pneumonia. Therefore, each risk factor was sorted out for Phase 4. Some additional risk indicators were suggested, but these were not used for Phase 4 due to the restricted frequency of the suggestions.

### Phase 4

The five characteristics of (nursing home-acquired) pneumonia and five risk indicators for considering the diagnosis aspiration pneumonia which had been selected in Phase 3, were presented to the 70 participants of Phase 2. Due to the fact that only 44 of them had responded in Phase 3, 15 additional experts were invited for strengthening a widely supported consensus. These 15 experts, who had not been recognised as such by the authors previously, were recommended by a geriatrician who was not involved in this Delphi survey. All invitees were requested to determine whether the five characteristics of (nursing home-acquired) pneumonia and the five risk indicators for considering the diagnosis aspiration pneumonia selected in Phase 3 were the most important ones, and whether at least three characteristics should be present to diagnose (nursing home-acquired) pneumonia adequately. For this purpose, a new questionnaire was made, containing ten questions: three multiple-choice questions, three structured open-ended questions and four open-ended questions. The multiple-choice questions were *‘Do you agree that the characteristics dyspnea, fever, deterioration of general functioning, tachypnea and crepitation with auscultation are the most important characteristics for diagnosing pneumonia in nursing home residents or do you have a deviating opinion?’, ’Do you agree that at least three of these characteristics should be present to diagnose nursing home-acquired pneumonia adequately or do you have a deviating opinion?’* and *‘Do you agree that the risk indicators dysphagia, choking incident, (history of) tube feeding, neurological disease and cognitive impairment in nursing home residents with pneumonia make you consider the diagnosis aspiration pneumonia or do you have a deviating opinion?’.* Structured open-ended questions were: *‘What is your medical specialty?’, ‘What is your age?’, ‘What is your gender?’.* The open-ended questions were *’Which of the following characteristics are not important when diagnosing pneumonia in nursing home residents?’,* ‘*Which characteristics are lacking for diagnosing pneumonia in nursing home residents?’, ’Which of the following risk indicators are not important for considering the diagnosis aspiration pneumonia in nursing home residents with pneumonia?’* and ‘*Which risk indicators are lacking for considering the diagnosis aspiration pneumonia in nursing home residents with pneumonia?’.*

Forty-four invitees completed the questionnaire: 24 men with an average age of 53.4 ± 5.6 years and 20 women with an average age of 49.4 ± 6.0, 36 elderly care physicians (82 %), 5 geriatricians (11 %), 2 pulmonologists (5 %) and 1 internist specialised in geriatrics (2 %).

Twenty-five participants (57 %) agreed that the characteristics dyspnea, fever, deterioration of general functioning, tachypnea and crepitation with auscultation are the most important characteristics when diagnosing (nursing home-acquired) pneumonia. Ten participants (23 %) mentioned the following characteristics as not important: fever (4), deterioration of general functioning (3), crepitation with auscultation (2) and dyspnea (1). Eight participants (18 %) mentioned that at least one important characteristic was lacking and five of them mentioned coughing as the most important lacking characteristic. The following additional characteristics were each mentioned once: delirium, feeling sick, change of behaviour, loss of appetite, sputum, nausea and vomiting. Due to the restricted frequency of these additional characteristics, they were not used any further. No consensus was reached about the presented characteristics for diagnosing (nursing home-acquired) pneumonia, because the consensus level was 57 % instead of the required 70 %. Although the required consensus level was not reached, the results suggested that dyspnea, fever, deterioration of general functioning, tachypnea and crepitation with auscultation are characteristics of some importance when diagnosing (nursing home-acquired) pneumonia.

To the question whether three characteristics should be present to diagnose (nursing home-acquired) pneumonia adequately, fifteen participants (34 %) agreed, thirteen (29 %) reported that the number of characteristics present is not relevant, two (5 %) reported that more than three characteristics are needed and 14 (32 %) reported that two characteristics should be present. Consensus was not reached, because only 34 % reported that at least three characteristics should be present. Summarising these data, the assumption is that two or three characteristics should be present for diagnosing (nursing home-acquired) pneumonia.

To the question whether the presence of the risk indicators dysphagia, choking incident, (history of) tube feeding, neurological disease and cognitive impairment make professionals consider the diagnosis aspiration pneumonia, 35 participants (80 %) agreed, yielding the required consensus of at least 70 %. Six participants (14 %) reported that a risk factor was lacking. Additional risk indicators suggested were: poor oral hygiene care, not responding to antibiotics, being in (sub)coma, auscultation in right lung and fever. Each of these risk indicators were mentioned only once and due to this restricted frequency, they were not used any further. Three (6 %) participants reported that cognitive impairment is not important when considering the diagnosis aspiration pneumonia. Eight participants mentioned that aspiration pneumonia cannot be diagnosed definitively on the basis of some risk indicators present, but that specific diagnostic tests are required.

## Discussion

Among the panel of clinical medical experts in geriatrics and pulmonology, no consensus was reached about the characteristics required for diagnosing (nursing home-acquired) pneumonia and about the number of characteristics required for diagnosing (nursing home-acquired) pneumonia. However, dyspnea, fever, deterioration of general functioning, tachypnea and crepitation with auscultation seem characteristics of some importance and the assumption is that two or three characteristics should be present. Subsequently, very satisfying consensus was reached about the risk indicators required for considering the diagnosis aspiration pneumonia in nursing home residents with pneumonia.

A potential explanation for not reaching consensus about characteristics required for diagnosing (nursing home-acquired) pneumonia and about the number of characteristics required, is that (nursing home-acquired) pneumonia usually presents itself by a wide variety of characteristics in different combinations [16]. The assumption derived from the current survey, namely that two or three characteristics should be present for diagnosing (nursing home-acquired) pneumonia, is consistent with the finding of a previous survey, being that patients with (nursing home-acquired) pneumonia had three or less respiratory or general characteristics [35].

In the current survey, the participants indicated fever as an important characteristic for diagnosing (nursing home-acquired) pneumonia. This is in contrast with the results of a previous survey, which showed that fever may only occur in 50 % of the older people with CAP [39]. In frail, very old people with pneumonia, fever was absent very often [40]. Subsequently, another survey demonstrated that less than half of a group of nursing home residents with pneumonia had either fever or a cough. In addition, 25 % of them had no clinical characteristics consistent with pneumonia at all [41]. These findings suggest that fever is a less important characteristic for diagnosing (nursing home-acquired) pneumonia than indicated by the participants of the current survey.

Although no consensus was reached about the (number of) characteristics required for diagnosing (nursing home-acquired) pneumonia, no additional expert-rating iterations have been performed because more than three expert-rating iterations do not usually yield improvement of concurrence [32]. For considering the diagnosis aspiration pneumonia in nursing home residents with pneumonia, a consensus level of 80 % was reached about the risk indicators dysphagia, choking incident, (history of) tube feeding, neurological disease and cognitive impairment, confirming the results of a recent literature review [42]. Considering these risk indicators, the commonly accepted definition that aspiration pneumonia is an infective pneumonia in a patient with a predisposition for aspiration [8], is confirmed. The presence of one or more risk indicators mentioned in the current survey may help to diagnose aspiration pneumonia at an early stage or even help to prevent aspiration pneumonia. To prevent nursing home residents from developing aspiration, swallowing screening and, in case of signs of dysphagia, further assessment by video endoscopic assessment of swallowing function are recommended [43–45]. Several studies have already indicated that oral hygiene care is an important aspect of preventing the various types of pneumonia [45–49]. More research is needed to determine which oral hygiene care interventions are the most effective in preventing aspiration pneumonia [20].

Some participants of the current survey expressed that aspiration pneumonia cannot be diagnosed without additional diagnostic tests. It has been suggested to use the available information of medical history and physical examinations, and to examine blood and sputum samples. Solid arguments for carrying out laboratory tests are that it is often difficult to identify medical history data and clinical characteristics in nursing home residents and that laboratory tests generally provide accurate information [50, 51]. To confirm the diagnosis definitely, chest radiographic examination with fluoroscopy is required [8, 11]. However, such examination is seldom possible in nursing homes.

NHAP is also defined as the presence of respiratory symptoms supported by abnormal findings in chest radiographs, such as an ill-defined shadow, consolidation or pleural effusion [52]. However, diagnosing NHAP only based on these findings may result in a falsely positive diagnosis, leading to an improper prescription of antibiotics. A decision not to perform radiographic chest examination has to be weighted with the risk of overtreatment with antibiotics. Furthermore, it has been suggested not to treat NHAP as a nosocomial infection, because infection with multidrug resistant bacteria is uncommon in NHAP. Only nursing home residents with severe pneumonia or high risk of infection due to multidrug resistant bacteria should be treated with a broad-spectrum antibiotic [53]. In addition, in a nursing home, it is generally not possible to confirm NHAP by chest radiographic examination and transferring ill nursing home residents to a hospital for such an examination is contra-indicated [12, 13]. In case a chest radiograph is considered necessary to confirm the diagnosis, it is feasible to use mobile radiography in the nursing home to avoid a burdening hospital transfer [54]. Finally, conversations in relation to ‘advance care planning’ elucidated that many nursing home residents want to be treated for pneumonia, but do not want to be referred to a hospital for diagnosis or treatment [55].

To determine if the selected risk factors are valid predictors for aspiration pneumonia, a clinical study is needed. Several experts mentioned that chest photos are needed to confirm aspiration pneumonia. A clinical study, whereby the diagnosis aspiration pneumonia made based on these risk factors will compared to diagnosis based on chest photos, will clarify if these risk factors are valid predictors for aspiration pneumonia. If so, using these risk factors lowers hospital costs, because less radiographic examinations are needed. Particularly because of the atypical presentation of (nursing home-acquired) pneumonia, it is recommended to observe nursing home residents accurately in order to notice any alternations. A previous survey reported that physicians who are frequently present in a nursing home perform less chest radiographs in cognitively impaired old people with pneumonia. Due to their frequent presence, these physicians had already noticed an alternation of the physical or cognitive status of the nursing home resident, indicating the onset of pneumonia [56]. When compared to physically disabled older people, pneumonia in cognitively-impaired older people presents itself even more with atypical non-respiratory characteristics, such as confusion and delirium [51]. Maybe these atypical characteristics can be noticed if the Care Dependency Status (CDS) of a resident by using a Care Dependency assessment is determined. This CDS allows nursing staff to observe subtle changes in this status,to manage care and provide better tailored care for individual residents [57].

Early diagnosis promotes the recovery of nursing home residents with pneumonia, because hospitalization due to serious pneumonia increases the frailty of nursing home residents [51]. Moreover, older people recovering from pneumonia are hospitalized for a longer time and recover more slowly, resulting in higher health care costs [14, 58]. A previous study found that hospital costs associated with aspiration pneumonia were more than fivefold those of non aspiration-related pneumonia [59]. Prevention of and early diagnosing of pneumonia reduce related costs, optimise health and promote quality health care [48, 60]. Most importantly, preventing pneumonia maintains the quality of life of nursing home residents [61].

The current survey had some limitations. Firstly, with regard to the expert panel and the response rates to the two general expert-rating iterations (Phases 2 and 3) and the consensus expert-rating iteration (Phase 4). Efforts were made to invite a reasonable number of experts. However, it was very difficult to select experts and the response rate was rather low. Secondly, an external validity hampering factor was that the expert panel was rather homogeneous, because predominantly experts in geriatrics in The Netherlands participated in Phase 4 (95 %) [62]. Consequently, the results of the expert panel are probably not representative for all medical experts in geriatrics. International consensus about the characteristics of NHAP has not been reached. In addition, the organisation of care, settings of nursing homes and the population of nursing home residents vary internationally. In The Netherlands, nursing homes contain somatic and psychogeriatric wards for primarily physically disabled residents and primarily cognitively impaired residents respectively [63–65]. Due to these international differences in care dependency in nursing homes, the results of this study may not be generalized without taking these differences into consideration. Therefore, it is recommended to consider further research on the risk factors of aspiration pneumonia in other countries than The Netherlands in order to reach international consensus. If further research is done, the specific definition of a nursing home and a long-term care facility should be taken into account.

## Conclusions

No final consensus could be reached among a panel of clinical medical experts in geriatrics and pulmonology on which and how many characteristics are required for diagnosing pneumonia in nursing home residents. However, the results indicated that dyspnea, fever, deterioration of general functioning, tachypnea and crepitation with auscultation are characteristics of some importance and that at least two or three characteristics should be present. For considering aspiration pneumonia in nursing home residents with pneumonia, final consensus was reached about the risk indicators dysphagia, choking incident, (history of) tube feeding, neurological disease and cognitive impairment.

## Abbreviations

CAP: community-acquired pneumonia
CDS: care dependency status
NHAP: nursing home-acquired pneumonia
